# Fabrication and Characterization of Silicon Micro-Funnels and Tapered Micro-Channels for Stochastic Sensing Applications

**DOI:** 10.3390/s8063848

**Published:** 2008-06-09

**Authors:** Marie J. Archer, Frances S. Ligler

**Affiliations:** U. S. Naval Research Laboratory, Center for Biomolecular Science and Engineering 4555 Overlook Ave SW, Washington D.C., 20375, USA; E-mail: frances.ligler@nrl.navy.mil

**Keywords:** stochastic sensing, silicon, anisotropic etching

## Abstract

We present a simplified, highly reproducible process to fabricate arrays of tapered silicon micro-funnels and micro-channels using a single lithographic step with a silicon oxide (SiO_2_) hard mask on at a wafer scale. Two approaches were used for the fabrication. The first one involves a single wet anisotropic etch step in concentrated potassium hydroxide (KOH) and the second one is a combined approach comprising Deep Reactive Ion Etch (DRIE) followed by wet anisotropic etching. The etching is performed through a 500 μm thick silicon wafer, and the resulting structures are characterized by sharp tapered ends with a sub-micron cross-sectional area at the tip. We discuss the influence of various parameters involved in the fabrication such as the size and thickness variability of the substrate, dry and wet anisotropic etching conditions, the etchant composition, temperature, diffusion and micro-masking effects, the quality of the hard mask in the uniformity and reproducibility of the structures, and the importance of a complete removal of debris and precipitates. The presence of apertures at the tip of the structures is corroborated through current voltage measurements and by the translocation of DNA through the apertures. The relevance of the results obtained in this report is discussed in terms of the potential use of these structures for stochastic sensing.

## 1. Introduction

Silicon micromachining techniques are widely used in various fields of engineering and biotechnology due to the possibility of fabricating structures with high aspect ratio and an extensive variety of geometries [1, 2]. Among them, dry and wet silicon etching techniques are, by far, two of the most important bulk micromachining processes. They are considered to have defined the beginning of micromachining and the MEMS discipline due to their relevance in the fabrication of MEMS and BioMEMS [3].

On a [100]-oriented silicon wafer, wet anisotropic etching through a square mask will produce pyramidal structures with a slope of 54.74° with respect to the (100) plane. This particular geometry results from the difference in the etch rates between the (111) and the (100) planes. The etch depth depends on the size of the mask opening; that is, the larger the mask opening, the longer it will take to reach the intersection of the (111) planes and the deeper the etch will be. Whether the structure formed has a sharp tip or a truncated pyramid will also depend on the etching time [1, 2, 4, 5]. The truncated pyramid structures leave a thin, un-etched region of silicon, commonly referred to as a membrane, and they can be made of silicon, silicon oxide or silicon nitride.

On the other hand, dry etching techniques, such as Deep Reactive Ion Etch (DRIE) do not rely on the etch rate of different crystallographic planes. The process uses a dry plasma of reactive species that interacts primarily with the exposed silicon surfaces while the sidewalls are protected from etching by a passivation layer. The process can be carried out at room temperature (Bosch process) or at cryogenic temperatures (-100 to -180 °C). The Bosch process consists of continuous switching between etching and passivation steps using SF_6_ (sulfur hexafluoride) as etchant and C_4_F_8_ (octafluorocyclobutane) as a passivation material. In the cryogenic process, the sidewall protection is driven by the formation of a blocking layer and by the reduction of the silicon surface reactivity due to the cryogenic environment [6, 7]. Structures with very high aspect ratios can be fabricated using these two techniques; the only difference is the smoothness of the trench sidewall, which is significantly enhanced in the cryogenic process.

Dry and wet etching techniques have been successfully combined to fabricate complex three-dimensional structures in silicon for microfluidics applications [8], electrical isolation on MEMS devices [9], and single-cell analysis [10]. Tapered out-of-plane nozzles, nanopipettes and needle-type arrays for various applications ranging from inkjet technology to tools for nanofabrication [11-17] have also been realized, but their fabrication is complex and requires multiple etching and deposition steps to acquire the desired out-of-plane characteristics, geometry, size and uniformity.

In this paper we present a fabrication approach that combines wet and dry etching of silicon to produce deep (∼500 μm) tapered structures using a single lithographic step and a silicon oxide hard mask. We envision the use of these structures as a possible alternative to the currently used solid-state nanopores for stochastic sensing. For this particular application, the ionic current through a single nanometer size aperture is measured in the presence of an analyte of interest (i.e. DNA or small molecules). The passage of the analyte through the nanopore will produce a characteristic current blockade that identifies its presence [18]. Currently single nanopores are fabricated on silicon or silicon nitride membranes using ion beam-enhanced etching or subsequent anisotropic etching, followed by narrowing through deposition or thermal oxidation [19-22]. Recently, it was suggested that having this nanopore at the tip of a tapered or conical structure, rather than in the center of a planar surface, would present three important advantages for stochastic sensing: 1) increased temporal resolution, 2) larger basal ionic currents and 3) higher detection sensitivity as the electric field is highly concentrated at the pore tip [23-24]. For instance, Mara and co-workers [23] recorded DNA translocation times in the order of miliseconds using conical nanopores, which is significantly slower than the microsecond duration events reported for cylindrical nanopores fabricated in silicon nitride membranes [20]. Also, due to geometrical differences, conical nanopores have lower ionic resistance than cylindrical nanopores; a difference of at least two orders of magnitude has been measured and this translates into an enhanced rate of analyte transport [25].

So far, tapered nanopores have been realized on polymer membranes using ion track etching [25-28] and on glass using wet chemical etching [29-31]. However, to the best of our knowledge, there is no documented approach on the fabrication of tapered nanopores in silicon.

**Figure 1.**
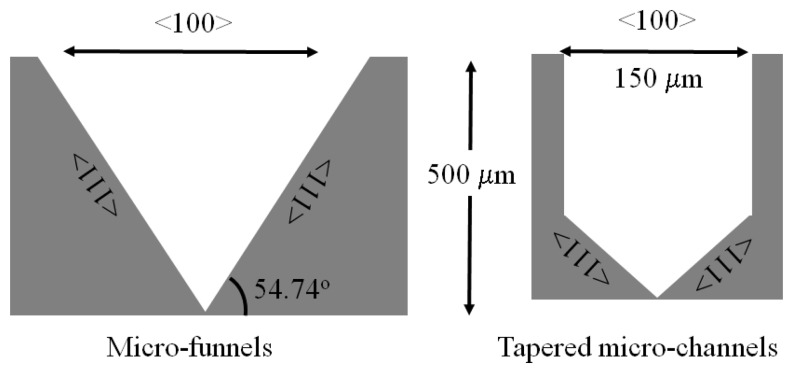
Schematic representation of the micro-funnels and tapered micro-channels.

In this paper we present the results of the first steps toward the fabrication of tapered structures, referred to as micro-funnels and tapered micro-channels, which could potentially be used in stochastic sensing. A schematic of the structures is shown in figure 1.

The differences in shape and dimensions could be used to study the effects of the diffusion and electric field distribution in the stochastic sensing of biomolecules or other analytes. An important motivation of the present work was to fabricate these structures in a simple and reproducible manner at wafer scale. For this purpose, we used a single lithographic step followed by either deep wet anisotropic etching or dry isotropic etching combined with wet anisotropic etching. We investigated the effect of the temperature and composition of the etchant, the characteristics of the hard mask, diffusion and micro-masking effects on the characteristics of the resulting layers. We also discuss the challenges in fabricating these particular types of structures at a wafer scale. The structures were analyzed through optical and scanning electron microscopy. Current-voltage (*I-V*) measurements and DNA translocation experiments were performed to asses whether an aperture was present at the tip, and we present an example of such results.

### 2. Results and Discussion

The motivation for this work was to investigate the possibility of fabricating, in a simple manner, at a wafer scale, tapered structures with sub-micrometer cross-sectional areas for potential use in stochastic sensing. For this purpose, we investigated a simplified process of fabricating micro-funnels and tapered micro-channels using a single lithographic step. The micro-funnels were fabricated using a single wet anisotropic etching step in 9 M KOH. The tapered micro-channels were fabricated using a combined approach of dry etching (Bosch process) to fabricate the shaft of the micro-channel followed by wet anisotropic etching in KOH to taper the end of shaft).

### 2.1. Effect of wet anisotropic etching conditions in the fabrication of micro-funnels

#### 2.1.2 Etchant Composition

Anisotropic etching of silicon comprises a continuous oxidation and dissolution process in which variables such as etchant composition, concentration, temperature and the presence of mediators affect the characteristics of the structures [4, 32-35]. An important parameter to consider is texturization of the surfaces characterized by the presence of micropyramids resulting from the re-deposition of the reaction products as well as the presence of H_2_ bubbles acting as “micro-masks” [34-37]. It is known that these artifacts can be minimized through the use of highly concentrated hydroxide solutions (up to 15 M) at temperatures up to 85 °C with or without the addition of isopropyl alcohol (IPA) [12, 35-36].

Since the fabrication of the micro-funnels required etching through the whole wafer, re-deposition of precipitates was an important concern. The first step was to optimize the etching conditions to minimize texturization and to produce uniform sidewalls. Although it is was not clear at this point how the micro-pyramids could affect the final shape of the tip, we expected that their presence on the sidewalls could interfere with the formation of the taper. For the intended purpose of these devices (stochastic sensing), anything interfering with the formation of the taper would affect the geometry of the tip and therefore the ionic current flow and the resistance. As a starting point for the optimization, we used the conditions published in the literature, which are 5-10 M KOH solutions at 80 °C with IPA addition. In a first set of experiments, rather than allowing the etching to proceed until the formation of a sharp tip, we evaluated the structures while a plateau was still visible at the bottom. All the structures fabricated with IPA resulted in texturized surfaces characterized by the presence of micro-pyramids on the bottom and the sidewalls of the structures. Whether these structures had an octagonal or a square base (hillocks) depended mainly on the etching time, but they were always present regardless of the hydroxide concentration.

Zubel and co-workers [36] observed this same effect in prolonged etches (3-5 hours) and attributed it to reaction products on the etched surface acting as micro-masks. In our case the etching was performed for a shorter period of time (∼1-2 hrs). Despite the fact that within this regime the addition of IPA has proven to produce smooth surfaces (even at low KOH concentrations) [35, 38], we were not successful in producing un-textured surfaces. We believe that the discrepancy with the published literature is due to an excess production of reaction products arising mainly from the dissolution of the silicon oxide mask, which was only 1 μm thick for this initial set of experiments. As the hard mask dissolves, the unprotected surfaces are also attacked by the etchant, increasing the amount of reaction products and favoring the conditions for the formation of micro-pyramids. This effect is enhanced as a larger surface is exposed since all the structures patterned on the wafer are contributing collectively to the effect. It is difficult to asses the specific role of IPA under these particular conditions; given the high production rate of reactants, their re-deposition might be dominating the processes making it very difficult for the few IPA molecules to maintain their moderating function enough to produce smooth surfaces.

In order to reduce the precipitate production rate and the dissolution of the silicon oxide mask without significantly compromising the smoothness of the structures, we investigated the effect of lower etching temperatures based on the following published information:

1) Surfaces comparable to those produced at 90 °C were obtained at lower temperatures if the etchant concentration was high enough. For instance etching at 70 °C in 10 M KOH (without added IPA) led to surfaces with smoothness comparable to those at 90 °C in 7-10 M KOH [35].
2) Micro-pyramid removal was attained by a short re-etch in the same bath at the same temperature (∼60-65 °C) where the first etch was done. Longer re-etching times led to new micro-pyramid formation [36].

These observations suggest that the etching temperature can be reduced without jeopardizing the surface uniformity if the KOH concentration is high enough, even without the addition of IPA. Since the etch rate of silicon oxide in KOH exhibits an exponential dependence on the temperature regardless of the concentration [32], a reduction of only 10-15 °C in the temperature can represent a significant enhancement in preserving the oxide mask and therefore reducing the production of reaction products. Also, the formation of micro-pyramids appears to be a “cyclic” process which depends on the etch rate of the crystallographic planes limiting the structures. Evidently, during a sufficiently long etch, micro-pyramids might appear and disappear as the structure tapers. If their occurrence has been minimized by reducing the rate of production of reaction products, their effect might be negligible after consecutive “re-etch” cycles.

#### 2.1.2 Effect of temperature and diffusion of reaction products

Based on the previous observations, we explored the possibility of using a lower etching temperature for the fabrication of the structures to reduce the texturization. Another important parameter that defines the surface uniformity is the diffusion of reaction products from the surface into the bulk solution. Zubel and co-workers [36] had suggested that vigorous stirring could be used to prevent their accumulation. In our case we had to ensure uniform diffusion across the whole wafer surface, and for this purpose, we placed a magnetic stir bar next to the wafer (lateral stirring) to facilitate the movement of reaction products. However, we did not observe any enhancement of the surface uniformity; instead we observed a more pronounced effect on the uniformity when the mask width was varied even without any stirring.

In order to test how a reduction in the amount of precipitates and the presence of different diffusion constraints would affect the uniformity, we performed an experiment using mask widths ranging from 600 μm to 1.3 mm. The etching was done at room temperature in 9 M KOH for 24 hours, and the structures were characterized using optical microscopy (results not shown). Although optical microscopy will not reveal detailed information about the wall smoothness or whether the artifacts are octagonal-based micro-pyramids or hillocks, it is an effective way to visualize large structural features and to address areas where the silicon oxide mask on the remaining surface might be compromised. The following observations were made:

1) The structures exhibited very uniform sidewalls, but there were evident inhomogeneities on the plateaus present as pits or agglomerates related to the width of the mask.
2) The presence of the pits tended to diminish with increasing mask widths, and the agglomerates were only present in the structures with the smallest widths.
3) Despite the fact that the thickness of the silicon oxide was only 1 μm thick, we did not observe any major disruption of the mask on the remaining surface.

Evidently, there is an effect of the width of the mask on the homogeneity of the structures. Diffusion of reaction products into the bulk solution and dislodging of H_2_ bubbles are facilitated in those structures with a larger width. In terms of the type of inhomogeneities observed, micro-pyramids are more likely to be a result of micro-masking by H_2_ bubbles or precipitates. The pits observed at the corners on the larger mask openings were similar to the circular or *doughnut*-shaped in-homogeneities described by Gonzalvez and coworkers and result from local variation in temperature and/or etchant concentration [34].

**Figure 2.**
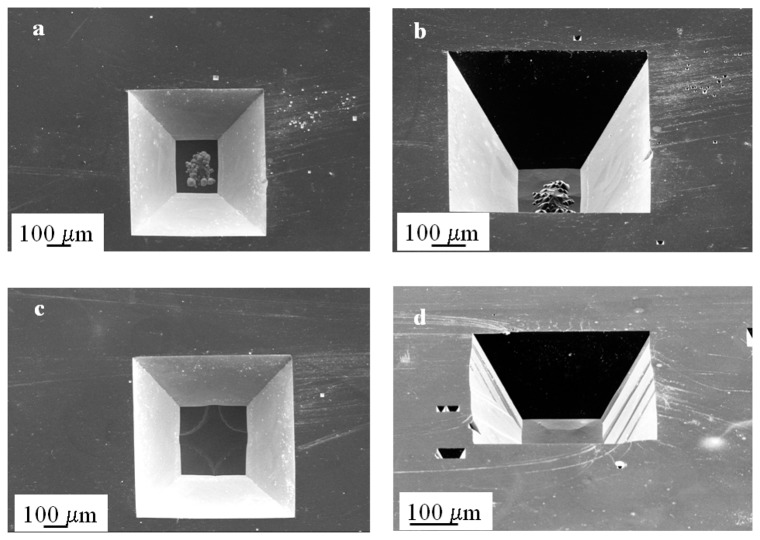
Scanning Electron Microscopy images of the structures fabricated with a 600 μm (a,b) and a 700 μm (c,d) square mask after re-etching in the same bath at 40 °C with addition of IPA to saturation. The presence of an agglomeration of hillocks at the bottom of the 600 μm mask opening as well as the circular pits at the bottom of the 700 μm opening are still evident.

In order to evaluate whether re-etching in the same solution would aid in the removal of the inhomogeneities, we immersed the wafer for an additional 24 hours in the same bath, this time heating to 40 °C. We added IPA to saturation to evaluate whether it would have any significant effect (positive or negative) on the surface texture. Figure 2 shows scanning electron microscopy images of the structures corresponding to the 600 and 700 μm mask width structures.

The remaining silicon oxide mask was stripped prior to scanning. These images show that the sidewall uniformity has been preserved and the oxide mask has been minimally compromised (as evident from the smaller pits on the sides caused by breakthrough of the etchant along localized areas on the mask). The agglomerate of hillocks in figure 2a-b (600 μm mask width) and the circular pits on the plateau in figure 2c-d (700 μm mask width) remained despite the increase in temperature and the addition of IPA. So far these results indicate that room temperature etching enhances the sidewall uniformity and helps preserve the oxide mask but does not eliminate the presence of texturization at the bottom of the structures. In this particular case, re-etching was not useful in smoothing the surfaces.

On the other hand, the lack of enhancement with lateral stirring might be due to the difficulty in maintaining a uniformly mixed layer over the whole surface of the 4” wafer. As described by Garcia and co-workers, as silicon dissolves in KOH, the viscosity near the surface increases, producing a layer known as “soluble glass” that changes the diffusion of reaction products into the bulk solution [39]. Lateral stirring might be displacing larger amounts of reaction products towards one side of the wafer, producing alterations in the local surface viscosity and favoring in-homogeneous etching. Etching under the same conditions using front stirring (stir bar placed perpendicular to the wafer plane separated by at least 1 cm) did not show any enhancement in the texturization of the surfaces and rather led to non-uniform etch rates along the wafer surface producing structures with pronounced steps or different depths. In this case the diffusion or reactants along the surface might also be affected by the pattern of the etchant flow.

#### 2.1.3 Optimization of conditions for deep anisotropic etching

**Figure 3.**
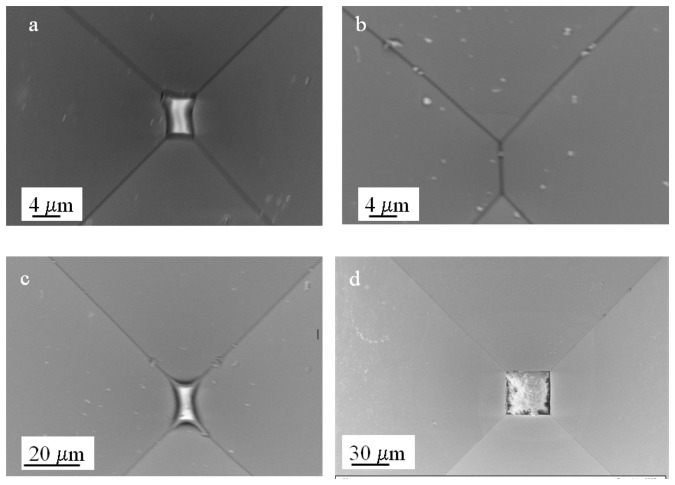
Scanning Electron Microscopy images of the tapered tip from structures fabricated with different mask openings sizes. (a) 400 μm, (b) 500 μm, (c) 600 μm and (d) 700 μm. Etching was performed in 9 M KOH at room temperature with no stirring. The etching was performed to a 500 μm depth.

In the experiments performed so far, the etching was stopped before the formation of a sharp tip, and this was useful to address the surface uniformity and the presence of texturization at the bottom of the structures. For stochastic sensing, a tapered end is necessary and this requires a longer etching time. As mentioned before, there is no documented information on how the inhomogeneities observed previously could affect the final shape and uniformity of the structures. Given the fact that these artifacts are due to diffusion of reactants which, as demonstrated before, is related in part to the width of the mask, we performed a set of experiments using mask widths from 700 to 400 μm (figure 3). Within this size range, etching for a certain period of time will produce structures with different depths and bottom plane widths (*Wo*). Thus we can evaluate whether micro-pyramid formation (if any) and/or precipitate trapping occurs at different etch depths and how it affects the final shape of the structures.

Since the formation of a tapered end would require a longer etching time, we used a 2 μm SiO_2_ mask to further prevent the excess production of reaction products. The wafer was immersed in a 9 M KOH solution and etched at room temperature for 96 hours without stirring and the electrolyte changed every 24 hrs. The change in the tip dimensions was followed using optical microcopy by imaging every 12 hours (results not shown). At the end of the etching period the structures corresponding to the 400, 500 and 600 μm mask widths had reached a “slit shaped” tip in most cases with dimensions between 2 and 6 μm in length (figure 3a-c). Despite the fact that there was no significant formation of micro-pyramids, we observed in some cases the presence of precipitates at the tip that were not necessarily related to the mask width (figure 3b).

The structure formed through the 700 μm mask produced plateaus with significantly variable dimensions and, in some instances, partial dissolution of the membrane (figure 3d). At first glance these results are not surprising since one would expect that, in a deeper structure with a narrow end, the diffusion constraints would be increased, “trapping” reaction products and bubbles, hence hindering the access to reactants and leading to non-uniform etching. A way to facilitate the diffusion of precipitants and the dislodging of H_2_ bubbles is to increase the temperature, which raised the question of how high could we go before running into the texturization issues observed previously. The optimization of this parameter was done by testing a range of temperatures below 80 °C and assessing the presence of inhomogeneities and/or precipitates through scanning electron microscopy. We found that a temperature between 50- 65 °C would produce structures with a negligible amount of precipitates at the tip, probably by facilitating diffusion and reducing the dwelling time of the H_2_ bubbles.

### 2.2 Assessment of fabrication reproducibility and uniformity of micro-funnels

To assess the uniformity and the reproducibility of micro-funnels fabricated with the optimized etching conditions, we patterned an array of 12 elements with 600 μm mask widths using a 2 μm SiO_2_ hard mask. The separation between each element was enough so that they could be cleaved to perform current-voltage (*I-V)* measurements and DNA translocation experiments on each to assess whether etch through the wafer had occurred. This also enables the characterization of each tapered structure as a single device, which is necessary for the intended stochastic sensing application. A practical way to evaluate the parameters influencing the etching was to include a row of 700 μm openings in the same pattern and to evaluate the presence of intact membranes, as well as their dimensions and degree of texturization as an indicator of the etching uniformity. Etching was performed for 24 hours in 9 M KOH at 65 °C without stirring. Scanning electron microscopy was used to characterize the structures.

**Figure 4.**
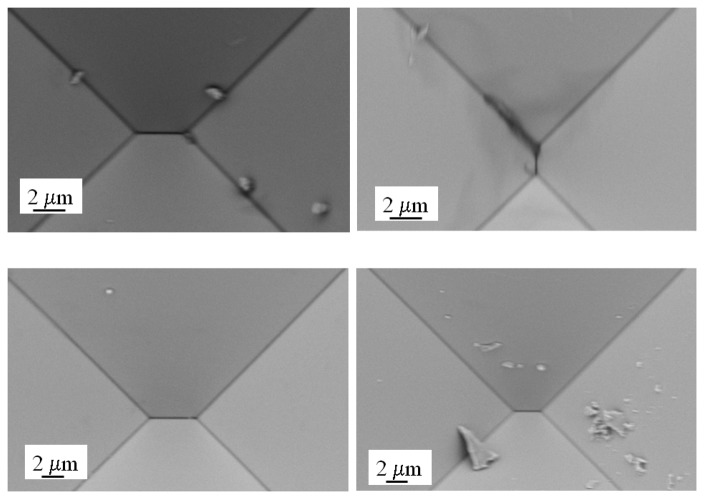
Scanning Electron Microscopy images of the tip of a representative set of micro-funnels fabricated with the optimized conditions (9 M KOH, 65 °C with no stirring). The openings were 600 μm width and the etching was performed to a 500 μm depth.

In terms of the shape, all the structures resulted in a slit-shaped tip; the average cross sectional area for structures fabricated using the 600 μm masks was 0.57 ± 0.13 μm^2^ in a sample size of 29 devices from 3 different wafers (confidence interval of 0.05). Three of the processed wafers were characterized using scanning electron microscopy and optical microcopy to obtain accurate quantitative information. Four additional wafers processed under the same conditions were characterized by optical microcopy and the devices obtained showed equivalent structural characteristics. Figure 4 shows a representative collection of scanning electron microscopy images of the tips from the structures fabricated through the 600 μm mask width.

**Figure 5.**
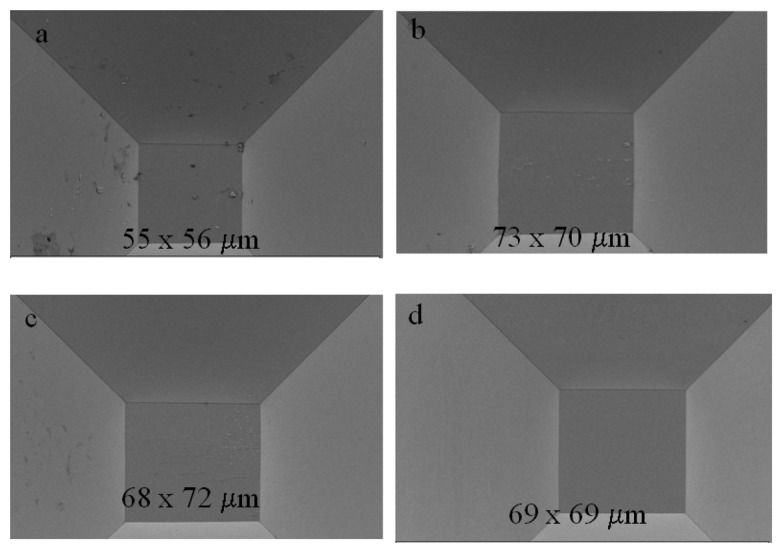
Scanning Electron Microscopy of the structures fabricated with the 700 μm square openings under the same conditions as the structures presented in figure 3.

Figure 5 shows the scanning electron microcopy images of the structures fabricated with the 700 μm mask openings on the same wafer and their dimensions. The uniformity of the 700 μm structures, the lack of texturization and the presence of intact membranes are an indicator of a “uniform” etching process, so the question that rises is what produces the variation in the cross sectional area of the tip? As mentioned in the previous section, diffusion is an important parameter, but it is probably more significant before the full development of the <111> planes. That is, as these planes become exposed, the etch rate is reduced and so is the production of reaction products and H_2_ bubbles. Thus the effect of such factors might be less significant as the depth increases. A second possible cause for the observed non-uniformities would be that the mask openings are not a perfect square but rectangles. Even if this was the case, as the depth increases, the characteristics of the mask aperture become less significant and the fully developed <111> planes become the dominant factor in the final geometry of the structure [1]. Furthermore, if a geometrical effect was to blame, the same elements would develop inhomogeneously in every wafer processed, which was not the case. A third possible cause would be the spatial distribution on the wafer. That is, when processing whole wafers, the uniformity of the structures would tend to diminish at the perimeter, but this was not observed either.

The results obtained can be explained based on the crystal's characteristics itself. Non-uniformities observed in deep anisotropic etching (above 500 μm) have been attributed to the presence of oxygen impurities present in the wafer as a result of the crystal growth process [38]. Crystal imperfections might be also responsible for the observed non-uniform tips. As it will become clear in the following section, these two effects along with diffusion become more significant in the fabrication of the tapered micro-channels. As mentioned before, the fact that the structures fabricated from the 700 μm square mask opening show uniform plateaus with similar areas regardless of their location supports the idea that the non-uniformities in the tips might be due to impurities and crystal orientations rather than other factors such as geometry, temperature gradients or local changes in etchant concentration.

### 2.3 Assessment of the presence of through-holes in micro-funnels

**Figure 6.**
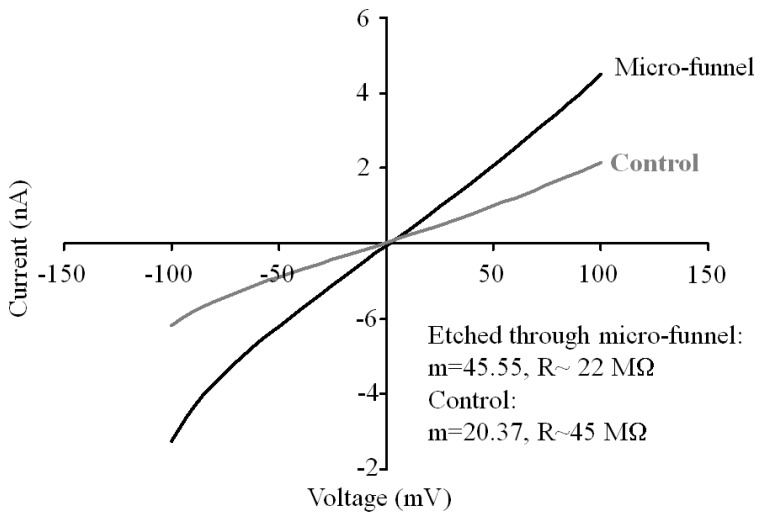
Characteristic current-voltage (*I-V)* curve obtained from a single micro-funnel that etched through the wafer during the anisotropic etching. The light gray line corresponds to the measurement from a solid silicon piece obtained from the same wafer. The difference in the slope indicates that an ionic current is flowing though the tip of the micro-funnel.

In order to evaluate whether the tapers had reached the bottom of the silicon and produced an aperture, we performed current-voltage (*I-V*) measurements in all of them. On average, no more than 50% of the structures had etched through. This assessment was performed by comparing the value of the resistance calculated from the slope of the *I-V* curve to the one obtained with a solid piece of silicon from the same wafer that was subject to the same acid cleaning treatments as the micro-funnel structures. *I-V* measurements have been used previously to determine the presence of a pore on a silicon substrate through a change in the ionic resistance [21]. Figure 6 shows a characteristic *I-V* curve from a 500 μm thick micro-funnel that etched through and a control (solid silicon) for comparison. The difference in the slope between both curves is clear. The lower resistance value of the micro-funnel with respect to the control indicates that there is an ionic current is flowing through the tapered tip. The values of resistance in M Ω calculated for the devices were 49.09 for the reference (solid silicon) and 21.4, 15.14, 17.43, 27.44, 28.77, 21.99, 47.09, 45.68, 48.79, 35.8, 40.2, 43.08 for the remaining 12 structures. The first six values differ in at least 20 MΩ with respect to the reference value while the last six are within a 10 to 1 M Ω difference suggesting that the likehood of an aperture is larger for the first group that the second one. For the first group there is variation in the resistance despite the fact that the observed dimensions of the tips are within a defined range (representative group in figure 4). This variation is related to the onset of the “punch through” of the tip during the anisotropic etching and will be discussed in section 2.4. We believe that the sizes of the apertures are close to or below 400 nm, since we were not able to use light to locate them in the taper.

In order to evaluate whether the published mathematical models could be used to determine the size of the apertures based on the experimental values of resistance, we calculated the dimension of the aperture for the first six values of resistance after re-baseline, with respect to the reference value, using the equation for the resistance of an electrolyte-filled conical nanopore [27]:

(1) $$R=\frac{\rho L}{\pi r_{\text{tip}}(r_{\text{tip}}+L\tan\theta )}$$

where R is the measured resistance in ohms; ρ is resistivity of the electrolyte, in this case 10 mM Tris-HCl at pH 8.5, (0.014 (Ω-m)^-1^ [42]); L is the length of the pore (500 μm); θ is the cone half angle (54.7°) and r_tip_ is the radii of the aperture. The value of r_tip_ is obtained by solving equation 1 for r_tip_ and obtaining the first root of the quadratic equation. Using these parameters we obtained values ranging from 400 nm up to 1 μm for the aperture diameter. However, we remain skeptical that these theoretical dimensions are applicable. We have attempted to measure current through single micron-sized through-holes on these structures, which are easily observable by electron microscopy, and this commonly leads to saturation of the instrument due to the large ionic current flow. A more feasible scenario would be that more than one nanometer-sized aperture is present in the membrane at the tip of the conical pore. There are several factors that have to be considered when analyzing the above discussed data. For instance, the model used has been developed for structures with a circular base and tip; in our case the base is square and the tip is “slit-shaped”; therefore work is needed to develop a more suitable geometrical model. Also, there is very little information published on the value of ionic conductance of the buffer used for these experiments; no more than two or three references are available, and the data are not consistent due to variation in experimental conditions [40-42]. It is worth emphasizing that, 10 mM Tris-HCl was selected as electrolyte as opposed to the more standard 1 M KCl, since it is the most suitable for the DNA translocation experiments that will be described in the following paragraph. Finally, parasitic currents due to exposed silicon surfaces might be contributing to the observed overall current. We have addressed this issue by minimizing the exposed silicon area though the design of the test chamber and by the use of a reference sample with identical structural characteristics (micro-funnel with no aperture).

**Figure 7.**
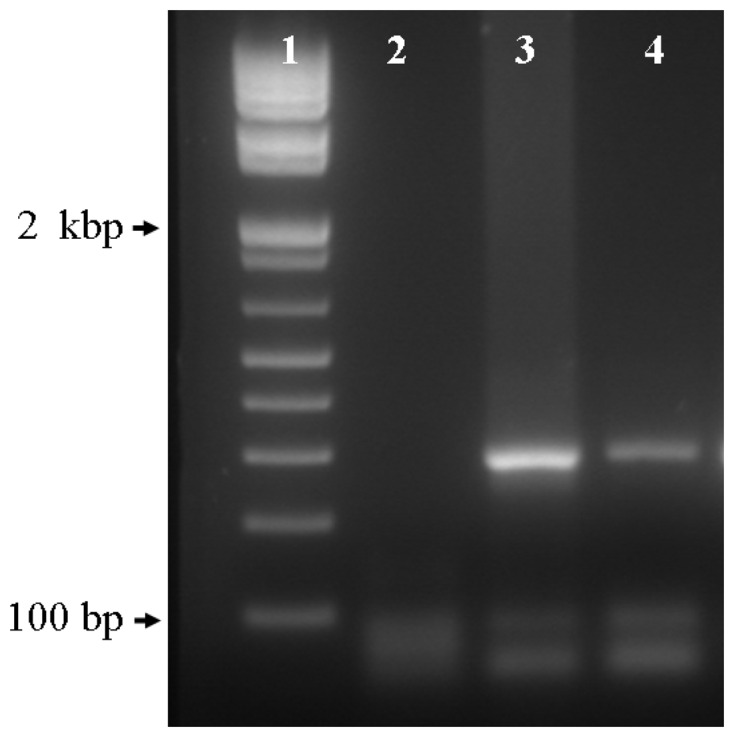
Representative image of the PCR products from the collected human DNA after translocation through a micro-funnel structure using a -500 mV potential. Lane 1, molecular weight marker, lane 2, PCR negative control; lane 3, PCR product from the remanent DNA collected at the cathode (-); lane 4, PCR product from the DNA that translocated from the cathode and was collected at the anode (+).

To further corroborate the presence of an aperture at the tip of the micro-funnels, we translocated human DNA using a constant potential of -500 mV. We tested two devices from the first group and one from the second group. It is worthwhile to emphasize that the purpose of these experiments was not to obtain real-time measurements of DNA translocation but to corroborate the presence of an aperture at the tip of the structures. Figure 7 shows a representative image of the PCR products on the recovered DNA at the cathode and the anode sides of a device with a resistance of 15.14 MΩ after 11 hours. Quantification of the collected products through UV/VIS spectrophotometry showed that only 10 % of the DNA passed through the aperture. The extensive time required for the translocation of DNA is probably due to the time required for diffusion of the genomic targets to the aperture site, but the specific mechanism underlying this lengthy process is out of the scope of the present study. No amplification was observed from the collected products using the high resistance devices indicating the lack of an aperture. Since there was no drift on the current in repetitive measurements over extensive period of time, the possibility of leakage around the devices was eliminated. The presence of an amplifiable target at the anode side demonstrates that DNA is translocating through the aperture at the tip of the micro-funnel. These results corroborate that the change in resistance observed with the *I-V* measurements corresponds to the presence of an aperture at the tip of the micro-funnels.

### 2.4 Tapered micro-channel fabrication and characterization

**Figure 8.**
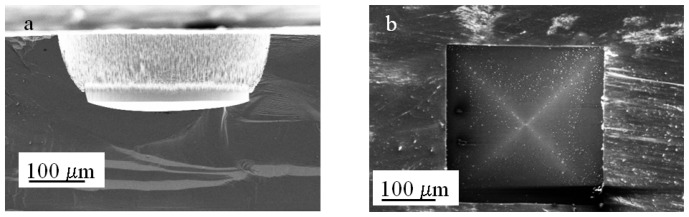
(a) Cross-section Scanning Electron Microscopy image of a structure fabricated using two consecutive deep reactive ion etch steps. The bottom width corresponds to the original mask width (150 μm) but the entrance of the structure has been widened. (b) High contrast scanning electron microscopy image of a micro-channel fabricated by four consecutive deep reactive ion etch steps (Bosch) followed by anisotropic etching in KOH. The lines defining the ribs of the inverted pyramid extend from each corner to the bottom of the taper suggesting that the four delimiting walls are at 54.7° with respect to the plane.

In the structures described previously, deep anisotropic etching of silicon enabled the fabrication of tapered structures with sidewalls at an angle of 54.7° with respect to the plane. We tested an alternative method to fabricate deep structures with tapered ends by combining dry and wet etching techniques, that is, deep reactive ion etch (DRIE) to produce a deep, straight channel followed by etching in KOH to taper its end. With this approach, the depth of the taper will correspond to the remaining silicon thickness, and therefore the mask width has to be smaller. In terms of the fabrication, this represents a larger barrier for the diffusion of reactants and reaction products as well as release of H_2_ bubbles. The difference in geometry with respect to the micro-funnels will also have an impact on stochastic sensing. One would expect that a narrower channel will change the resistance as well as the electric field distribution and therefore the movement of analytes. The fabrication of the tapered micro-channels was performed using either two consecutive steps: one 300 μm DRIE followed by a 50 μm DRIE (referred to as two-step etch) or three 100 μm DRIE steps followed by a 50 μm deep one (referred to as four-step etch). We had previously observed that a “sequential” etch approach would render channels with “stacked” openings of different width (figure 8a) which could be beneficial for diffusion of the etchant in and out the structure.

An advantage of using the Bosch process rather that a cryogenic one is that the former produces rippled sidewalls which we believe facilitates the wetting and therefore the infiltration of the etchant. The structure presented in figure 8a was originally fabricated using a 400 μm mask width, using two 100 μm deep sequential Bosch etching steps. Evidently, the second step produced a wider entrance, but the bottom of the structure preserved the dimensions of the original mask, which means that the depth of the taper will be defined by the patterned width size.

**Figure 9.**
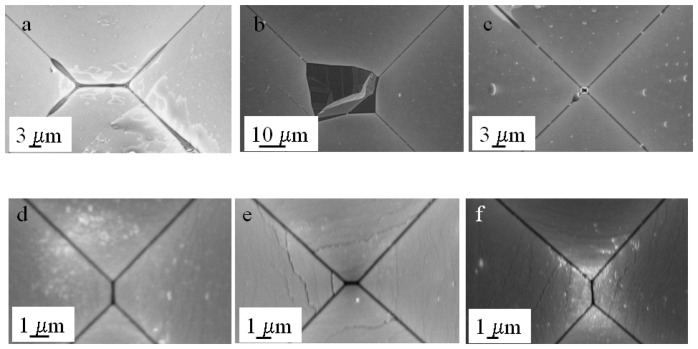
Scanning Electron Microscopy images of a collection of tapered micro-channels fabricated using (a-c) two consecutive steps of deep reactive ion etching (Bosch) followed by anisotropic etching to taper the tip. The diffusion constraints produced by the narrow geometry channel produces non-uniformities and residue accumulation at the tip. The use of four consecutive steps (d-f) leads to a wider channel entrance that facilitates diffusion, producing tapers with a more uniform size range and geometry.

For the experiments, an array of 16 square elements with aperture widths of 150 μm was patterned on a 4 inch wafer with a 2 μm SiO_2_ mask. The prepared substrates were subject to the two-step or the four-step etch as previously described, cleaned with an O_2_ plasma and immersed in 9 M KOH at 65 °C for 8 to 10 hrs. The devices prepared with the two-step etch exhibited a maximum increase at the channel entrance of 10-20 μm from the original width (150 μm). In comparison the devices prepared with the four-step etch doubled the size of the original width. In terms of the uniformity of the shape and the size of the tapers, the two-step approach produced mainly “slit shaped” tapers with lengths between 1 and 11 μm. We observed that some of the structures exhibited irregular shapes and large solid silicon fragments (figure 9 a-c).

The four-step approach also produced slit-shaped tapers but with a narrower size range between 1 and 3 μm in length and ∼ 200 nm width. In this case, we did not observe major disturbances in the shape or dimensions of the tapers (figure 9 d-f). In either case no more than 50 % of the tapers led to an aperture as corroborated by current-voltage *(I-V)* measurements. It is worthwhile emphasizing that the size of the taper does not necessarily correspond to the size of an aperture. It is possible that one or more apertures could be present along a single slit; multiple apertures have been observed in other slitlike structures [22]. For these devices the resistance range was wider, probably resulting from the wider range in the slit dimensions and the likehood of more than one aperture. The range was 1MΩ to 33.3 MΩ for the etched devices in comparison with the reference device of 33.53 MΩ. Fifty percent of the devices exhibited a difference of at least 15 MΩ with respect to the reference, and DNA translocation experiments were performed using two of these devices. As before, the presence of an aperture was corroborated by amplification of the collected products on the anode through the polymerase chain reaction (results not shown).

The sequential etch approach produces structures with stacked openings of different sizes. As the anisotropic etching proceeds and the (100) surfaces exposed at the interface between each stack are etched, the sidewalls of the channels become limited by the (111) planes. Thus, it would be expected that the final shape of the channel would follow the same geometry, that is, layers but with walls at 54.7° with respect to the plane. Surprisingly, the four-step approach produced sidewalls delimited by the (100) planes that extended from the entrance of the channel to the bottom. Figure 8b shows a high contrast scanning electron microscopy image of one of the structures. The ribs of the structure are evidenced by the brighter lines that extend from the upper corners to the bottom of the structure. We did not observe this pattern in the two-step approach, indicating that the observed tapering is more pronounced at the bottom of the structure and the shaft of the micro-channel remains straight.

The wider size range, the irregular shapes, and the presence of silicon fragments in the structures fabricated with the two-step approach are in part due to diffusion constraints. That is, reactants, reaction products and H_2_ bubbles will have a more difficult time moving through the channel. The widening of the channel entrance in the four-step approach seems to reduce the diffusion constraints as evident from the narrower size range and the lack of irregular structures. Despite this, we also observed the presence of residues but mainly in the form of crystals. We believe that, in either case, the presence of oxygen impurities and crystal imperfections are responsible for the “slit shaped” tapers as well as their size range.

The diffusion constraints discussed for the two-step etch structures affect their fabrication and use for stochastic sensing of analytes present at very low abundance. It would be expected that these constraints will increase the time required for the analytes to reach the tip of the structure. For this particular application, the structures fabricated with the four-step etch approach would be better suited since diffusion might be facilitated by the widening of the channel entrance. The difference in geometry with respect to the micro-funnels provide a testbed for the investigation of the geometrical effects on diffusion and electric field distribution.

### 2.5 Opening of the tapered micro-funnels and micro-channels and potential applications in sensing

The possible use of the taper structures as devices for stochastic sensing requires the taper to have an open end. As discussed before, no more than 50% of the structures punched through during the tapering step. The wide range of resistance values (despite the defined range of cross sectional areas at the tip) suggests that the aperture is not complete along the slit. The irreproducibility on the etch through during the first etching step could be due to changes in the etching behavior at the tip of the structure as a result of oxygen impurities. Holke and co-workers [43] reported non-uniform etching and reduced etching rate as a result of oxygen impurities during deep anisotropic etching of narrow channels in concentrated KOH solutions. This could explain the lack of consistency in the aperture of the tapered structures. The other issue that has to be considered for practical applications is the shape of the tapered end (tip) since certain applications might require a symmetric shape rather than a slit.

We are currently working on methods to address these fabrication issues. An important parameter that is also being considered is to account for the variations on the wafer thicknesses that result from the manufacturer tolerance. In terms of the taper shape, we have observed that the structures preserve the square shaped bottom to a certain depth and, as the etching proceeds, they become slits. We are investigating approaches to fabricate the aperture at the point where the bottom width is still square shaped.

### 2.6 Dry vs. wet etching of the hard mask and implications in the uniformity

One of the issues that significantly affected the uniformity and reproducibility of the etched structures was the process used to open the silicon oxide hard mask. In a first set of experiments, we used reactive ion etch (RIE) to open the silicon oxide mask, and we observed that the etching was not uniform along the wafer. Inspection through optical microcopy and profilometry measurements confirmed this issue. Despite the fact that the wafer was placed in the center of the etching chamber, the etch rate always appeared to reduce towards one edge of the wafer. Longer processing times to ensure the complete removal of the oxide layer would eventually etch through the resist layer in some areas therefore compromising the oxide hard mask. The use of a thicker resist layer would allow for a longer etching time to ensure a complete opening of the mask but with the risk of resist degradation [44]. For the case of the tapered micro-channels that have to undergo a DRIE process, extended etching is definitely a detrimental factor and would require a second lithographic step. Compromising the silicon oxide mask leads to non-uniform etching along the elements patterned on the wafer and thinning of the silicon frame of the structures after KOH etching, making the structures fragile and difficult to handle. Since the etch rate of silicon oxide in KOH is much lower than that of silicon, the etching will proceed non-uniformly within the same opening, leading to sidewall flaking and non-reproducible tapers.

**Figure 10.**
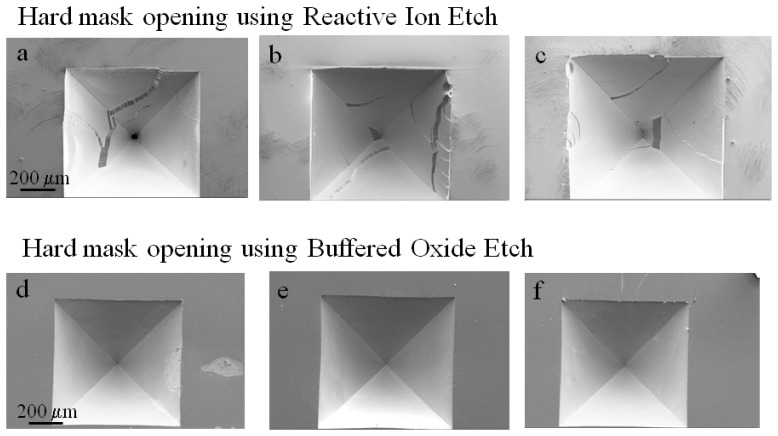
Scanning Electron Microscopy images of a set of structures in which the mask opening was performed using reactive ion etch (a-c) and buffered oxide etch (d-f) after wet anisotropic etching with KOH. The non-uniform etch of the silicon oxide mask using reactive ion etch reduces the sidewall smoothness and the reproducibility of the tapered structures. The etching was performed to a 500 μm depth.

In comparison, a chemical etch using a buffered HF solution (BOE) produced uniform dissolution of the oxide mask along the whole wafer. Profilometry measurements and optical microscopy on each element patterned on the wafer revealed that the resist layer was not affected during the process and that the 2 μm of oxide were completely dissolved. Figure 10 shows a set of scanning electron microscopy images of structures after KOH etching under the optimized conditions (9 M KOH at 65 °C) using a reactive ion etch (RIE) and buffered oxide etch (BOE) for the mask opening.

### 2.7 Oxide mask stripping and residue formation

One of the issues that have to be taken into account when fabricating the deep tapered structures, especially those with narrow entrances, is the possibility of residue accumulation. This is of critical relevance when the presence of an aperture has to be corroborated through current-voltage measurements as well as for subsequent use for stochastic sensing. We observed the accumulation of large amounts of residue at the bottom of the structures after removal of the SiO_2_ hard mask. Despite the fact that the structures were extensively rinsed with MilliQ water after anisotropic etching, a very distinctive type of residue was found at the tip of the tapers.

**Figure 11.**
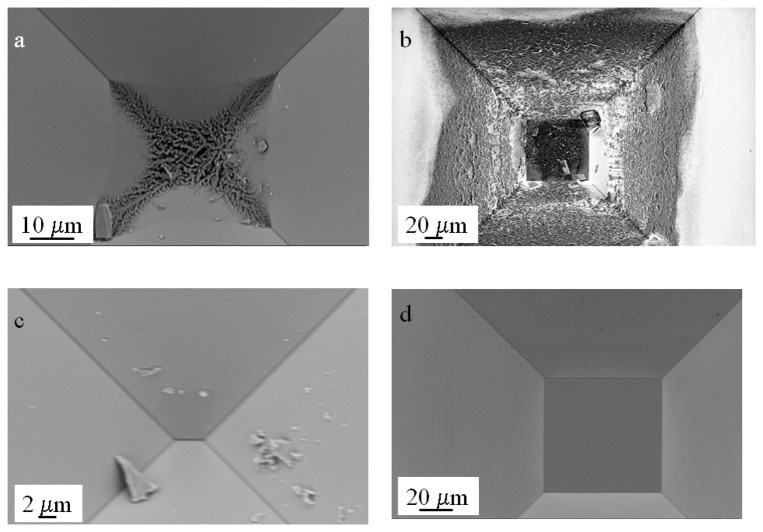
Scanning Electron Microscopy images of devices after stripping the silicon oxide mask and abundant rinsing with MilliQ water (a-b). Residue is present and accumulates at the bottom of the taper and on the plateau. The same devices after cleaning with 25% HF in ethanol and storage in 50% ethanol (c-d).

Figures 11a and 11b show a collection of scanning electron microscopy images of structures subject to this treatment. The residue observed at the bottom of the structures is not likely to be due to environmental particles since all these processes were performed in a laminar hood inside a class 100 cleanroom. The presence of these residues is not observable immediately after KOH etching but rather after the oxide mask stripping, suggesting that re-deposition of the etched material is taking place or that residues from the anisotropic etchant are reacting with the hydrofluoric acid. The crystals observable in figure 11b originate from the KOH solution or from the presence of impurities in the KOH pellets (i.e. Pb). This issue is of significant relevance in stochastic sensing when assessing the presence of apertures in the tapered structures since the crystals may block the current flow and produce inconsistent results. Furthermore, the presence of even small particles at the tip produce a blockade in the current as they occlude the aperture at the tapered end. We found that the most effective way to clean the structures after removal of the hard mask was to immerse them in a 25% HF ethanolic solution with lateral stirring for 20 minutes, rinse them thoroughly with ethanol and store them in 50% ethanol in an air tight container until scanning. Figures 11c and 11d show scanning electron microscopy images of the same structures after cleaning.

## 3. Experimental Section

### 3.1 Mask design

Two different masks were used to fabricate micro-funnels. The first one comprised rows of four square apertures of different sizes from 700 to 400 μm, and it was used to investigate the effect of the mask opening size on the final dimensions and shape of the tapered end. The second one consisted of 12 square apertures of 600 μm and four 700 μm apertures, and it was used to fabricate an array of micro-funnels and to evaluate their uniformity and reproducibility at the wafer scale. As will become clear in the following sections, the 700 μm row is used as an indicator of the etching uniformity. The expected etch depths for each mask aperture were calculated according to:

(2) $$W_{o}=W_{m}-\sqrt{2}*z$$

where:

*Wm*, mask width (μm)
*Wo*, bottom plane width (μm)
*z*, etch depth (μm)

Considering a bottom plane width of ∼ 1 μm, the depth of the structures would vary between 495 μm for the 700 μm mask to 282 μm for the 400 μm mask. However, as it will become clear in the discussion section, the accuracy of these numerical calculations is confounded given the multiple variables involved in the etching process. The variability is also increased by variation in the thickness of the wafer (+/- 25 μm) and the large etching area. The calculations were used only as a guide to select a range of mask openings that would provide incremental etch depths. For the tapered micro-channels, we selected a mask width of 150 μm. Assuming that the channel shaft produced by deep reactive ion etch (DRIE) would be 300-350 μm and that the etch depth (*z*) is ∼0.707 times the mask width, the overall taper would be ∼ 116 μm deep, which would bring the taper tip close to the bottom of the wafer.

### 3.2 Fabrication

**Figure 12.**
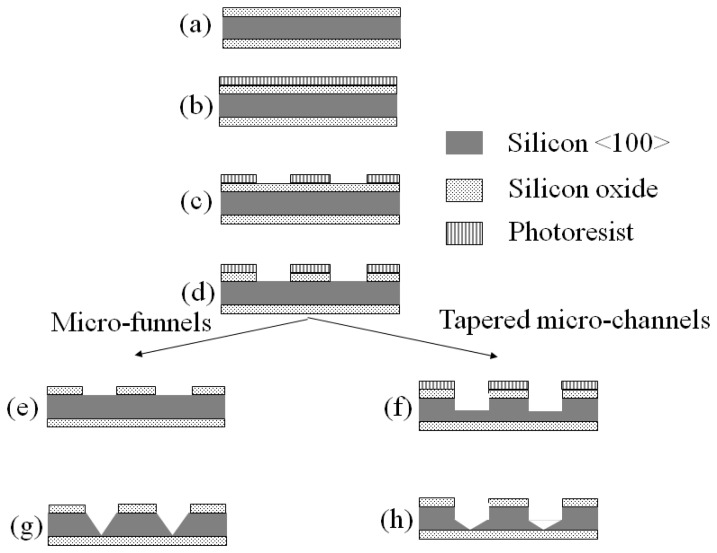
Process flow. The substrate used is a <100> p-type silicon wafer with a silicon oxide layer of 1 or 2 μm. Patterning is performed using standard lithographic steps.

The two fabrication approaches are schematically shown in figure 12.

All structures were fabricated on single-side polished, 100 mm diameter, p-type boron-doped (10-100 ohm-cm) <100> silicon wafers, 500 +/- 25 μm, with either 1 or 2 μm thermally grown silicon oxide (SiO_2_) (Surface Process Group, Richmond VA). Photolithography was performed on the polished side of the wafer using AZP4330 resist (Clariant, Co. Ltd) or Shipley 1818 (Shipley, Marlborough, MA) according to the manufacturer recommendations. The silicon oxide mask was opened using reactive ion etcher (Axic Benchmark 800) at 30 mT, 175 W, 30 sccm with a mixture of CF_4_ (96%) and nitrogen (4 %) for 30-45 minutes or chemically using buffered oxide etch solution (10:1) (BOE) at room temperature for 40 minutes. Step-height analysis was performed before and after the mask opening through contact profilometry (Alpha Step IQ, KLA Tencor Co.) Deep reactive ion etch (Oxford Plasma Lab, Oxford Instruments) was used to etch 300 μm deep channels using a standard Bosch process in two or four consecutive steps for a final depth of ∼350 μm. The wafers were cleaned under an oxygen plasma at 30 mT, 175 W, 30 sccm for 15 minutes. Anisotropic etching was performed in 9 M KOH with or without isopropyl alcohol (IPA) added to saturation in a temperature range between 22 and 80 °C. The silicon oxide mask was stripped using a 25% HF aqueous solution. The wafers were cleaned with a 1:1 (v/v) solution of hydrochloric acid and methanol, rinsed with MilliQ water and then immersed in a 50% ethanol before imaging. Optical microscopy and scanning electron microscopy were used to characterize the structures.

### 3.3 Current-voltage measurements and DNA translocation experiments

Prior to current-voltage (*I-V*) measurements, the etched wafers were cleaved to provide individual devices with one structure per device, treated with Piranha etch (H_2_SO_4_: H_2_O_2_ 3:1 v/v) at 70 °C for 30 min to ensure a hydrophilic surface, and stored in ethanol until use. For characterization, the devices were mechanically fixed between two Teflon chambers, each with a 7 mm^2^ window, using a thermosensitive polymer on each side to ensure a proper seal. The micro-funnels were positioned at the center of the open window of the chamber to ensure a complete exposure to the electrolyte, and the chambers were filled with 200 μl of TE buffer (10 mM Tris, 1mM EDTA, pH 8.0) containing 1% Tween 20. Only the area corresponding to the 7 mm^2^ window was exposed to the electrolyte; the surrounding silicon along with the cleaved edges was isolated from the electrolyte by the polymer film. A voltage was applied across the devices using Pt wire electrodes, and an Axopatch 200B Integrating Patch clamp amplifier (Axon Instruments Inc.) was used to control the voltage and record the output current. The current was allowed to stabilize for at least 20 minutes prior to performing the *I-V* measurements. For the DNA translocation experiments, a voltage of -500 mV was applied across the device, and 10-20 μg of human DNA were added to the cathode side. Passage of DNA through the aperture was allowed to proceed for 11 hours to ensure a maximum collection on the anode side. To avoid settling of the DNA on the cathode side, the electrolyte was re-suspended periodically by pipeting. The volume on the anode side was collected and concentrated using ethanol precipitation, quantified through UV/VIS sprectrophotometry, and analyzed by direct visualization on an ethidium bromide-stained 1.2% agarose gel or by performing PCR using the GADPH gene as a target sequence. The PCR products were then electrophoresed on a 1.2% ethidum bromide-stained agarose gel against a positive control to corroborate the specific amplification of the target sequence.

## 4. Conclusions

The use of solid nanopores for stochastic sensing applications has several advantages in comparison with biological pores (e.g. α-hemolysin) such as stability, ease of chemical functionalization and reusability. For this reason there has been an increasing interest to develop methods for fabricating single nanopores on solid supports. Solid nanopores have been successfully realized in different substrates such as polymers, silicon nitride and silicon oxide membranes and metals using techniques such as ion beam sculpturing and ion track etching, however they require specialized equipment and their fabrication can be very challenging [18, 21-23]. It would therefore be desirable to produce these devices in a simple and reproducible manner at a larger scale. In an effort to address this challenge, in this work, we presented a process to fabricate arrays of tapered micro-funnels and micro-channels using deep anisotropic etching of silicon at a wafer scale. The resulting structures are characterized by wide entrance delimited by four planes that intersect in a slit-shaped taper with sub-micron cross sectional area. The influence of the electrolyte composition, temperature, stirring and mask quality were discussed in terms of the shape and the size of the taper. We envision the use of these devices for stochastic sensing of small molecules (e.g. proteins, viruses, explosives), and for this purpose the presence of a through-hole at the slit-shaped tapered is of critical relevance. To demonstrate that the proposed fabrication approach renders devices with a through-hole we performed current-voltage (*I-V*) measurements and corroborated these quantitative results by translocating DNA though the apertures. The movement of DNA from the cathode to the anode across the aperture in the presence of an electric field was confirmed by performing PCR on the collected DNA at the anode. Our results demonstrate that a reduction in resistance with respect to a reference device (no aperture) indicates the presence of a through-hole. In average a through-hole was produced in 50% of the devices fabricated on a single wafer.

Currently we are testing other protocols to increase the number of devices with through-holes and elucidating the critical parameters that determine the production of an aperture. We have speculated that more than one through-hole could be present on the same slit, and we are currently pursuing other techniques to characterize the structures (Transmission Electron Microscopy). The functionality of the devices will then be address through stochastic sensing of viral particles.

## References

[1] Madou M. (1997). Fundamentals of Microfabrication.

[2] Kovacs G.T.A. (1998). Micromachined Transducers Sourcebook.

[3] Ziaie B., Baldi A., Lei M., Gu Y., Siegel R.A. (2004). Hard and soft micromachining for BioMEMS: review of techniques and examples of applications in microfluidics and drug delivery. Adv. Drug Deliver Rev..

[4] Bean K.E. (1978). Anisotropic Etching of Silicon. IEEE T. Electron Dev..

[5] Vaganov V. (2004). Method for fabrication microstructures with deep anisotropic etching of thick silicon wafers.

[6] Kovacs G.T.A., Maluf N.I., Petersen K.E. (1998). Bulk micromachining of silicon. P. IEEE.

[7] de Boer M.J., Gardeniers J.G.E., Jansen H.V., Smulders E., Gilde M.-J., Roelofs G., Sasserath J.-N., Elwenspoek M. (2002). Guidelines for etching silicon MEMS structures using fluorine high-density plasmas at cryogenic temperatures. J. Microelectromech. S..

[8] de Boer M.J., Tejerkstra W., Berenschot J.W., Jansen H.V., Burger G.J., Gardeniers J.G.E., Elwenspoek M., van der Berg A. (2000). Micromachining of buried microchannels in silicon. J. Microelectromech. S..

[9] Zhu Y., Yan G., Fan J., Zhou J., Liu X., Li Z., Wang Y. (2005). Fabrication of keyhole-free ultra-deep high-aspect-ratio isolation trench and its applications. J. Micromech. Microeng..

[10] Matthews B., Judy J.W. (2006). Design and fabrication of a micromachined planar patch-clamp substrate with integrated microfluidics for single-cell measurements. J. Microelectromech. S..

[11] Laurell T., Wallman L., Willson J. (1999). Design and development of a silicon microfabricated flow-through dispenser for on-line picolitre sample handling. J. Micromech. Microeng..

[12] Mukhopadhyay D., Ferreira P.M. (2007). Exploiting differential etch rates to fabricate large-scale nozzle arrays with protudent geometry. J. Micromech. Microeng..

[13] Guenat O.T., Generelli S., Dadras M., Berdondini L., de Rooij N.F., Koudelka-Hep M. (2005). Generic technological platform for microfabricating silicon nitride micro and nanopipette arrays. J. Micromech. Microeng..

[14] Huang H., Fu C. (2007). Different fabrication methods of out-of-plane polymer hollow needle arrays and their variations. J. Micromech. Microeng..

[15] Andersson H., van den Berg A. (2003). Microfluidic devices for cellomics: a review. Sensor. Actuat. B-Chem..

[16] Paik S.J., Byuna S., Lima J.-M., Park Y., Lee A., Chung S., Changa J., Chuna K., Choa D. (2004). In-plane single-crystal-silicon microneedles for minimally invasive microfluid systems. Sensors Actuat. A-Phys..

[17] Zahn J.D., Talbot N.H., Liepmann D., Pisano A.P. (2000). Microfabricated polysilicon microneedles for minimally invasive biomedical devices. Biomed. Microdevices.

[18] Schmidt J. (2004). Stochastic sensors. J. Mater. Chem..

[19] Schmidt C., Mayer M., Vogel H. (2000). A Chip-based biosensor for the functional analysis of single ion channels. Angew. Chem. Int. Ed..

[20] Li J., Gershow M., Stein D., Brandin E., Golovchenko J.A. (2003). DNA molecules and configurations in a solid-state nanopore microscope. Nature.

[21] Chen P., Mitsui T., Farmer D.B., Golovchenko J., Gordon R.G., Branton D. (2004). Atomic Layer Deposition to fine-tune the surface properties and diameters of fabricated nanopores. Nano Lett..

[22] Park S.R., Peng H., Ling X.S. (2007). Fabrication of nanopores in silicon chips using feedback chemical etching. Small.

[23] Mara A., Siwy Z., Trautmann C., Wan J., Kamme F. (2004). An asymmetric polymer nanopore for single molecule detection. Nano Lett..

[24] Harrell C., Choi Y., Horne L.P., Baker L.A., Siwy S.Z., Martin C.R. (2006). Resistive-pulse DNA detection with a conical nanopore sensor. Langmuir.

[25] Li N., Yu S., Harrell C.C., Martin C.R. (2004). Conical nanopore membranes. Preparation and transport properties. Anal. Chem..

[26] Heins E.A., Siwy S.Z., Baker L.A., Martin C.R. (2005). Detecting single porphyrin molecules in a conically shaped synthetic nanopore. Nano Lett..

[27] Scopece P., Baker L.A., Ugo P., Martin C.R. (2006). Conical nanopore membranes: solvent shaping of nanopores. Nanotechnology.

[28] Wharton J.E., Jin P., Sexton L.T., Horne L.T., Sherrill S.A., Mino W.K., Martin C.R. (2007). A method for reproducibly preparing synthetic nanopores for resistive-pulse biosensors. Small.

[29] Fertig N., Meyer C., Blick R.H., Trautmann C., Behrends J.C. (2001). Microstructured glass chip for ion-channel electrophysiology. Phys. Rev. E..

[30] White R.J., Zhang B., Daniel S., Tang J.M., Ervin E.N., Cremer P.S., White H.S. (2006). Ionic conductivity of the aqueous layer separating a lipid bilayer membrane and a glass support. Langmuir.

[31] Zhang B., Galusha J., Shiozawa P.G., Wang G., Bergren A.J., Jones R.M., White R.J., Ervin E.N., Cauley C.C., White H.S. (2007). Anal. Chem..

[32] Seidel H., Csepregi L., Heuberger A., Baumgartel H. (1990). Anisotropic etching of crystalline silicon in alkaline solutions i. orientation dependence and behavior of passivation layers. J. Electrochem. Soc..

[33] Seidel H., Csepregi L., Heuberger A., Baumgartel H. (1990). Anisotropic etching of crystalline silicon in alkaline solutions II. Influence of dopants. J. Electrochem. Soc..

[34] Gosalvez M.A., Sato K., Foster A.S., Nieminen R.M., Tanaka H. (2007). An atomistic introduction to anisotropic etching. J. Micromech. Microeng..

[35] Zubel I., Kramkowska M. (2001). The effect of isopropyl alcohol on etching rate and roughness of (100) Si surface etched in KOH and TMAH solutions. Sensor. Actuat. A-Phys..

[36] Zubel I. (1998). Silicon anisotropic etching in alkaline solutions II On the influence of anisotropy on the smoothness of etched surfaces. Sensor. Actuat. A-Phys..

[37] Schroder H., Obermeier E., Steckenborn A. (1999). Micropyramidal hillocks on KOH etched {100} silicon surfaces: formation, prevention and removal. J. Micromech. Microeng..

[38] Merlos A., Acero M., Bao M.H., Bausells J., Esteve J. (1993). TMAH/IPA anisotropic etching characteristics. Sensors Actuat. A-Phys..

[39] Garcia S.P., Bao H., Hines M.A. (2004). Etchant anisotropy controls the step bunching instability in KOH etching of silicon. Phys. Rev. Lett..

[40] Bakewell D.J.G., Hughes M.P., Milner J.J., Morgan H. (1998). Dielectrophoretic Manipulation of avidin and DNA. Proceedings of the 20th Annual International Conference of the IEEE Engineering in Medicine and Biology Society.

[41] Fullarton J.R., Kenny A.J. (1970). A rapid system for preparative electrophoresis depending on isoelectric buffers of low conductivity. Biochem. J..

[42] Hjerten S. (1995). Electrophoresis in low conductivity buffers.

[43] Holke A., Hendersson H.T. (1999). Ultra-deep anisotropic etching of (110) silicon. J. Micromech. Microeng..

[44] Walter L. (1997). Photoresist damage in reactive ion etching processes. J. Electrochem. Soc..

